# Liquid-phase milk turbidimetry enables rapid phenotyping of secreted protease activity in *Pseudomonas aeruginosa* at less than one cent per sample

**DOI:** 10.1128/spectrum.01271-26

**Published:** 2026-09-10

**Authors:** Karla Daniela Enríquez-Ríos, Juan Carlos Pineda-Licona, Selene García-Reyes

**Affiliations:** 1 Instituto de Química, Universidad Nacional Autónoma de México. Ciudad Universitaria 7180, Ciudad de México, Mexico; 2 Programa Investigadoras e Investigadores por México-Secihti, Mexico, Mexico; Institut National de Santé Publique du Québec, Sainte-Anne-de-Bellevue, Québec, Canada; Arla Foods Amba, Aarhus N, Denmark; Emory University, Atlanta, Georgia, USA

**Keywords:** *Pseudomonas aeruginosa*, secreted proteases, milk turbidimetry, quorum sensing

## Abstract

Secreted proteases are major virulence determinants in *Pseudomonas aeruginosa*. Their production varies widely across strains and quorum-sensing (QS) backgrounds, creating the need for simple, standardized methods to quantify extracellular proteolytic activity. Here, a liquid-phase skim-milk turbidimetric assay is described to quantify total secreted protease activity in clarified culture supernatants using a Tris–CaCl₂ buffer and turbidity loss at 600 nm (A600) as the spectrophotometric endpoint. Reactions were incubated at 37°C while shaking for 3 h before measurement. Reference strains PAO1 and PA14, a QS-deficient double mutant (PAO1Δ*lasR*Δ*rhlR*), and two additional isolates (DVL1758 and ATCC 9027) were evaluated under assay-matched growth conditions. Endpoint culture density was comparable across strains (OD600 ~ 3.4–4.0; one-way ANOVA, *P* > 0.05), supporting the interpretation of A600 differences as extracellular activity phenotypes. The assay resolved strain-dependent milk clearance at two practical substrate loads (2 mg and 5 mg), distinguishing low-, intermediate-, and high-proteolysis phenotypes, with the QS mutant exhibiting minimal clearance. Intralaboratory precision was acceptable for a biological assay (CV < 20% across strains and loads). Robustness across loads was supported by strong agreement between mean A600 values at 2 mg and 5 mg (Pearson *r* = 0.861, *P* = 0.0277, *R*² = 0.741; excluding medium control *r* = 0.954, *R*² = 0.910). This low-cost, widely accessible workflow enables rapid comparative profiling of secreted proteolytic phenotypes and complements more specific substrate-based or orthogonal validation assays when mechanistic resolution is required.

**IMPORTANCE:**

Understanding how bacteria produce enzymes that break down proteins is key to studying infection and developing new treatments. *Pseudomonas aeruginosa*, an important opportunistic pathogen, secretes proteases that contribute to tissue damage and disease progression. However, measuring total protease activity across strains remains technically challenging, costly, or difficult to standardize. Here, we present a simple, low-cost liquid-phase milk turbidimetry assay that enables rapid and accessible quantification of secreted protease activity using standard laboratory equipment. This method reliably distinguishes between strains with different proteolytic phenotypes, including quorum-sensing-deficient mutants, and demonstrates good reproducibility and robustness across conditions. Because it uses widely available reagents and requires minimal processing, this assay lowers barriers for routine phenotyping and large-scale screening. Our approach provides a practical tool for studying virulence, regulatory mechanisms, and potential anti-virulence strategies in *P. aeruginosa* and related organisms.

## INTRODUCTION

*Pseudomonas aeruginosa* is a clinically important opportunistic pathogen responsible for acute and chronic infections, particularly in patients with compromised host defenses or disrupted epithelial barriers (1–6). Its adaptability across environments and host tissues is supported by extensive regulatory circuitry and a large repertoire of secreted factors that promote colonization, persistence, and tissue damage (7–11).

Among extracellular virulence determinants, secreted proteases are key contributors to the pathogenicity of *P. aeruginosa*, promoting nutrient acquisition, tissue remodeling, and evasion of host immune defenses (9). These enzymes support nutrient acquisition and host tissue remodeling and can impair innate immune defenses by degrading structural proteins and immune-related components (9). For this reason, total secreted protease activity is widely used as an integrative functional readout that reflects the cumulative extracellular activity of multiple secreted proteases rather than the activity of any individual enzyme. As such, it provides a measure of the net extracellular proteolytic output under defined conditions and constitutes a biologically meaningful phenotype for strain comparison and functional studies (9, 12). The major secreted proteases include elastase LasB, alkaline protease (AprA), LasA protease, and protease IV (PIV) (9, 13–15).

The production of these proteases in *P. aeruginosa* is strongly regulated by quorum sensing (QS), which coordinates population density-dependent expression of numerous virulence factors, including secreted proteases (16). QS-deficient mutants display markedly reduced extracellular protease production (13), highlighting the importance of QS in virulence regulation and making this pathway an attractive target for antimicrobial strategies.

Accurate quantification of proteolytic activity is, therefore, essential for understanding the physiological roles of proteases, investigating regulatory mechanisms, and evaluating therapeutic interventions.

Multiple approaches have been used to quantify or detect total protease activity in *P. aeruginosa*. Skim-milk agar clearing assays offer simple qualitative or semi-quantitative screening but are influenced by diffusion and growth effects and are less suited to standardized, higher-throughput comparisons (12, 17). Liquid assays based on specialized substrates, such as elastin–Congo red, chromogenic casein derivatives (for example, azocasein), or fluorogenic peptides, can provide improved quantification, yet often trade accessibility for complexity through substrate availability, higher reagent cost, additional processing steps, or instrumentation requirements (12, 17). These constraints can hinder routine implementation and complicate cross-study comparisons when large, isolated panels are evaluated.

These limitations motivate a simple liquid-phase workflow that preserves biological relevance while improving accessibility and scalability. Milk proteins, dominated by caseins, provide a complex but widely available substrate that can capture broad proteolytic activity without relying on specialized synthetic reagents. In a liquid format, proteolysis can be tracked by turbidity loss, providing a direct spectrophotometric readout at visible wavelengths that could be readily adapted to standard microplate readers. Because the assay relies only on absorbance measurements at 600 nm, it could potentially be adapted to standard microplate readers for higher-throughput applications. A liquid milk turbidimetry format could, therefore, enable scalable, low-cost phenotyping of secreted proteolysis applicable to strains with distinct proteolytic phenotypes.

Here, a liquid-phase milk turbidimetric assay is presented to detect total secreted protease activity in *P. aeruginosa* culture supernatants using skim milk as the substrate in Tris–CaCl₂ buffer and turbidity loss at 600 nm (A600). The central hypothesis is that this method resolves QS-dependent and strain-specific protease phenotypes across reference strains, QS-deficient mutants, and clinical or environmental isolates, and that activity trends are concordant with established protease assays when comparisons are appropriately normalized for growth.

## MATERIALS AND METHODS

### Bacterial strains and growth conditions

The *P. aeruginosa* strains used in this study included the reference laboratory strains PAO1 (MPAO1 subline) and UCBPP-PA14 (PA14), as well as a PAO1-derived QS-deficient double mutant (PAO1Δ*lasR*Δ*rhlR*). To evaluate the assay’s ability to discriminate naturally occurring variation in secreted proteolysis, two additional isolates with distinct protease-associated phenotypes reported in prior studies were included: the environmental isolate *P. aeruginosa* DVL1758 and the clinical isolate *P. aeruginosa* ATCC 9027 (Table 1).

**TABLE 1:** List of *P. aeruginosa* strains used in this study

Strain	Description/genotype	Source/origin	Reference
PAO1	Wild-type reference strain	Infected wound isolate (Australia, 1950)	(18)
PAO1*ΔlasRΔrhlR*	PAO1-derived QS-deficient double mutant (*lasR rhlR*)	Laboratory-derived mutant	(19)
PA14	Wild-type reference strain	Burn wound isolate	(20, 21)
DVL1758	Environmental isolate	Shallow pond isolate	(22)
ATCC 9027	Clinical isolate	Outer-ear isolate	(23)

All strains were cultured in LB broth under the same conditions used for supernatant production for total protease activity determination. Endpoint culture density was assessed as OD600 after 16 h to confirm comparable biomass across strains.

### Bacterial growth assay

Overnight cultures of each *P. aeruginosa* strain were used to inoculate 5 mL of fresh LB broth to an initial OD600 of 0.05. Cultures were incubated for 16 h at 37°C with shaking at 220 rpm. Endpoint growth was quantified by measuring optical density at 600 nm (OD600) using a spectrophotometer. These endpoint OD600 measurements were used to compare bacterial growth across strains under the same experimental conditions used for the protease assay.

For total protease determination, 1 mL aliquots of each culture was clarified by centrifugation at 12,000 rpm for 5 min at room temperature. The resulting supernatants were collected without disturbing the pellet and used immediately for the milk turbidimetry assay.

### Milk substrate and reaction buffer preparation

The substrate used for the total protease assay was a 2% (wt/vol) skim milk solution prepared from skimmed milk powder (we used the commercial product: Svelty Move+ with Collagen, Nestle) in distilled water. The commercial skim milk powder used in this study was selected because it was readily available and provided consistent performance throughout assay development and validation. Because commercial milk formulations may differ in protein composition and additives among manufacturers or production lots, the same product and preparation protocol were used for all experiments. Users applying this assay are encouraged to standardize the milk source within a given study and, when changing brands, to perform a preliminary validation to confirm comparable assay performance.

The reaction buffer consisted of 0.1 M Tris–HCl (pH 7.0) supplemented with 1 mM CaCl₂. The skim milk solution and assay buffer were sterilized separately by autoclaving (121°C, 15 min) and cooled to room temperature before use. Although autoclaving may induce structural changes in milk proteins, including whey protein denaturation and alterations in casein micelle organization, sterilized skim milk is routinely used in protease-detection media. Because all assays were performed using substrate prepared under identical conditions, any heat-induced effects were standardized across experiments and were not expected to affect relative strain comparisons.

Calcium supplementation was included because divalent cations can stabilize and/or support the activity of extracellular proteases, including metalloprotease-associated activity commonly produced by *P. aeruginosa*, improving assay consistency.

### Protease activity assay

Total secreted protease activity was determined by liquid-phase milk turbidimetry using clarified culture supernatants. Reactions were prepared using a 2% (wt/vol) skim milk substrate (20 mg/mL) in Tris–CaCl₂ assay buffer (0.1 M Tris–HCl, pH 7.0, 1 mM CaCl₂). Two substrate loads were evaluated:

- **Condition A (2 mg milk per reaction):** 100 µL culture supernatant + 100 µL milk substrate + 800 µL assay buffer (final volume 1.0 mL). Final buffer composition is 80 mM Tris-HCl and 0.8 mM CaCl₂).
- **Condition B (5 mg milk per reaction):** 100 µL culture supernatant + 250 µL milk substrate + 650 µL assay buffer (final volume 1.0 mL). Final buffer composition is 65 mM Tris-HCl and 0.65 mM CaCl₂).

Reactions were incubated at 37°C with shaking (220 rpm) for 3 h. Residual turbidity was quantified as absorbance at 600 nm (A600) using a quartz cuvette. Although all measurements in this study were performed using quartz cuvettes, the assay chemistry is compatible with absorbance measurements in standard microplate readers after appropriate optimization of reaction volume and optical path length. Lower A600 values indicate increased milk clearance and higher apparent total secreted protease activity.

### Controls and strain panel

PAO1 and PA14 were included as high-protease reference strains, whereas the QS-deficient mutant PAO1Δ*lasR*Δ*rhlR* served as a negative protease reference. DVL1758 and ATCC 9027 strains were included to evaluate assay performance across additional strain backgrounds. A medium-only background control (LB control) consisted of milk substrate plus assay buffer supplemented with LB medium in place of culture supernatant and was used as the non-enzymatic turbidity reference.

### Statistical analysis

All statistical analyses were performed using Microsoft Excel and Origin (OriginLab). Endpoint growth (OD600) and milk turbidimetry readouts (A600) were analyzed by one-way analysis of variance (ANOVA). When a significant strain effect was detected, group means were compared using Tukey’s honestly significant difference (HSD) post hoc test (*α* =0.05) to enable multiple pairwise comparisons across all strains. Three independent biological cultures were prepared for each strain. Each culture supernatant was assayed in triplicate (technical replicates), and the mean technical value was used for statistical analysis. Statistical significance was defined as *P* < 0.05.

To evaluate robustness across substrate loads, mean A600 values obtained at 2 mg and 5 mg milk loads were compared by Pearson correlation (two-tailed) and simple linear regression (least squares) in Origin software. Regression models were fit with A600 at 5 mg as the dependent variable and A600 at 2 mg as the independent variable; slope, intercept, and *R*² are reported. Analyses were performed both including and excluding the LB medium-only control, as indicated.

### Reproducibility assessment (intralaboratory variability)

Intralaboratory reproducibility of the milk turbidimetry assay was assessed under both substrate loads (Condition A: 2 mg; Condition B: 5 mg) using three independent biological replicates per *P. aeruginosa* strain. For each replicate, residual turbidity was quantified as absorbance at 600 nm (A600). The mean and standard deviation (SD) were calculated, and precision was summarized as the coefficient of variation (CV%), computed as:


$$CV(\%)=\left(\frac{SD}{mean}\right)\times 100$$

CV values < 10% were interpreted as high intralaboratory reproducibility, whereas CV values between 10% and 20% were considered acceptable for biological assays. All calculations were performed using Microsoft Excel.

## RESULTS

### Growth comparison among *P. aeruginosa* strains

**Fig 1:**
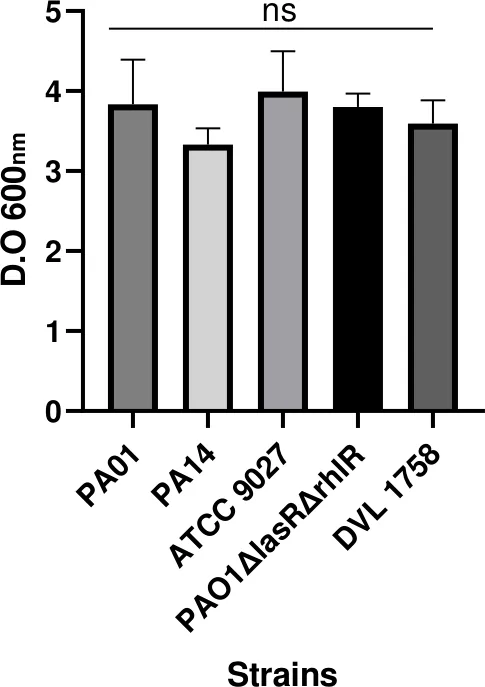
Comparable endpoint growth across *P. aeruginosa* strains under assay-matched conditions. Strains were grown in LB for 16 h, and endpoint density was measured as OD600. Data are presented as mean ± SD of three independent biological replicates, each measured in triplicate technical replicates. No differences were detected among strains (one-way ANOVA, *P* > 0.05; ns), indicating that subsequent differences in milk turbidity (A600) are not attributable to biomass variation.

To ensure that differences in milk degradation were not attributable to variation in bacterial biomass, endpoint growth was assessed under the same conditions used to generate culture supernatants for the total protease assay. After 16 h incubation in LB broth, all strains reached comparable final densities (OD600 ≈ 3.4–4.0; Fig. 1), and one-way ANOVA detected no significant differences among groups (*P* >0.05). Controlling for growth is a common requirement when comparing secreted virulence-associated products across *P. aeruginosa* strains because extracellular protease readouts are often normalized to culture density (OD600) or total protein to distinguish true secretory phenotypes from biomass-driven effects. Accordingly, the strain-dependent patterns observed in the milk turbidimetric assay are unlikely to be explained by differential growth and instead support genuine differences in secreted protease activity. Therefore, A600 differences were interpreted without additional OD normalization because endpoint OD600 values did not differ significantly among strains (Fig. 1), indicating comparable final culture densities. To further evaluate whether the modest variation in biomass could influence the interpretation of protease activity, a sensitivity analysis was performed by normalizing residual turbidity values to the corresponding mean endpoint OD600. The results of this analysis are presented after the characterization of the protease assay (Table 3).

### Milk turbidimetry distinguishes total protease phenotypes

**Fig 2:**
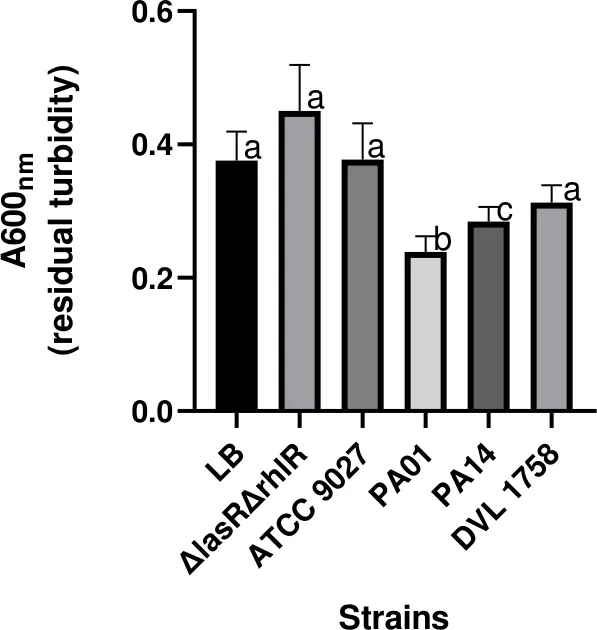
Strain-dependent milk clearance at low substrate load (2 mg). Clarified supernatants were incubated with skim milk (2 mg) in Tris–CaCl₂, and residual turbidity was read as A600.Data are presented as mean ± SD of three independent biological replicates, each measured in triplicate technical replicates. Different letters indicate significant differences (one-way ANOVA + Tukey’s HSD, *α* = 0.05); lower A600 indicates higher protease activity.

When the assay was performed with a 2 mg skim milk load, clear strain-dependent differences in residual turbidity were resolved (Fig. 2; Table 1). The LB medium control, the QS-deficient mutant (PAO1Δ*lasR*Δ*rhlR*), ATCC 9027, and DVL1758 showed the highest A600 values (0.38 ± 0.04, 0.45 ± 0.07, 0.38 ± 0.05, and 0.31 ± 0.03, respectively), indicating minimal substrate clearance and no-protease phenotype under these conditions. In contrast, PAO1 and PA14 exhibited markedly lower turbidity (0.24 ± 0.02 and 0.28 ± 0.02), consistent with enhanced milk degradation and higher secreted total proteolytic activity. Although DVL1758 has previously been reported to produce extracellular proteases (24), including LasB, it clustered with the low-protease group under the 2 mg substrate condition, likely reflecting limited assay resolution at the lower substrate load

The strain-dependent differences in milk clearance are biologically consistent with the established role of QS in regulating extracellular protease production in *P. aeruginosa*. Disruption of QS is well known to reduce secreted protease output, including elastase-associated activity, providing a mechanistic basis for the high-turbidity/low-degradation signal observed for the PAO1Δ*lasR*Δ*rhlR* mutant in this assay. Skim-milk-based readouts, whether agar clearing or liquid-phase turbidity loss reported here, are widely used as practical proxies for comparative “total protease” phenotyping across strains and regulatory backgrounds. Together, these results indicate that the 2 mg condition provides strong discriminatory power for separating low and high total protease phenotypes while maintaining acceptable intralaboratory precision (Table 2).

**TABLE 2:** Quantitative residual turbidity (Mean A600 ± SD) and intralaboratory reproducibility (CV%) of the liquid-phase milk turbidimetry assay under the 2 mg and 5 mg substrate-load conditions^*a*^

Strain	Mean A600 ± SD (2 mg)	CV % (2 mg)	Mean A600 ± SD (5 mg)	CV % (5 mg)
LB control	0.38 ± 0.04^a^	11.63	1.77 ± 0.09^a^	4.99
PAO1Δ*lasR*Δ*rhlR*	0.45 ± 0.07^a^	15.35	1.64 ± 0.25^a^	15.14
ATCC 9027	0.38 ± 0.05^a^	14.44	1.19 ± 0.22^b^	18.07
PAO1	0.24 ± 0.02^b^	9.75	0.67 ± 0.05^c^	8.12
PA14	0.28 ± 0.02^c^	7.72	0.71 ± 0.10^c^	13.96
DVL1758	0.31 ± 0.03^a^	8.44	0.69 ± 0.11^c^	15.22

^*a*^ Data are presented as mean ± SD of three independent biological replicates, each measured in triplicate technical replicates. Superscript letters indicate significant differences among strains within the same milk load (one-way ANOVA with Tukey HSD, *α *= 0.05). For confirmatory statistics, analyzes should be repeated using replicate-level data.

**Fig 3:**
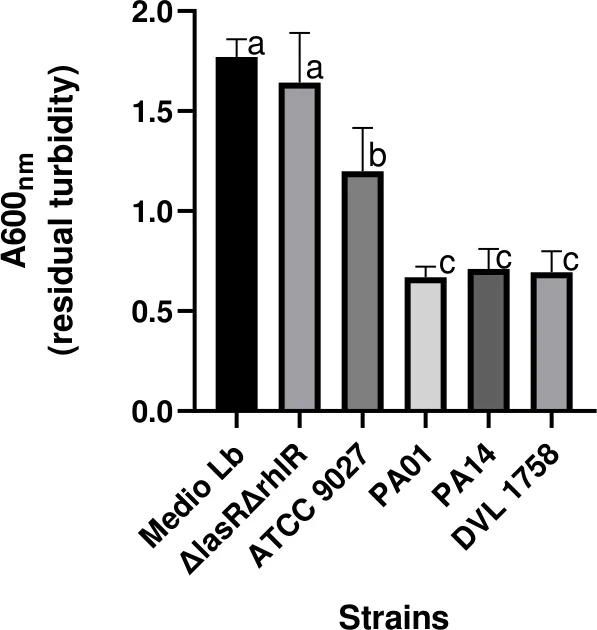
Strain-dependent milk clearance at higher substrate load (5 mg). Clarified supernatants were incubated with skim milk (5 mg) in Tris–CaCl₂ and residual turbidity was measured as A600. Data are presented as mean ± SD of three independent biological replicates, each measured in triplicate technical replicates. Different letters indicate significant differences among strains (one-way ANOVA + Tukey’s HSD, *α* = 0.05); lower A600 indicates higher protease activity.

A similar strain-dependent pattern was observed at the higher substrate load (5 mg; Fig. 3; Table 1), with higher absolute A600 values reflecting the increased amount of milk protein present in the reaction. The LB medium control and the QS-deficient mutant (PAO1Δ*lasR*Δ*rhlR*) again showed the highest residual turbidity (1.77 ± 0.09 and 1.64 ± 0.25, respectively), consistent with minimal milk clearance and a low total protease phenotype. The ATCC 9027 strain displayed an intermediate A600 value (1.19 ± 0.22), indicating limited but detectable substrate degradation relative to the negative controls.

In contrast, PAO1, PA14, and DVL1758 strains produced the lowest A600 values (0.67 ± 0.05, 0.71 ± 0.10, and 0.69 ± 0.11, respectively), consistent with robust milk clearance and higher apparent total secreted protease activity. Overall, the strain ranking observed at 5 mg was consistent with that obtained at the 2 mg substrate load, supporting the interpretation that the assay captures stable differences in extracellular proteolysis rather than load-dependent artifacts.

Notably, DVL1758 displayed an intermediate behavior across substrate loads. Under the 2 mg condition, its residual turbidity values fell between those of the highly proteolytic strains (PAO1 and PA14) and the low-activity group, resulting in less distinct statistical separation. At 5 mg, however, the increased substrate load improved signal resolution, and DVL1758 clustered with PAO1 and PA14 while remaining clearly separated from the low-protease phenotypes. This shift in statistical grouping occurred without altering the overall ranking of the strains and, therefore, likely reflects improved analytical discrimination rather than a change in the underlying biological phenotype.

This behavior is consistent with prior evidence that QS regulates major extracellular proteases in *P. aeruginosa*, such that QS-deficient backgrounds typically show reduced proteolytic phenotypes in milk-based readouts. Collectively, the 5 mg condition provides a higher baseline turbidity while preserving strain ranking, supporting assay robustness across substrate loads and its utility for comparative protease phenotyping.

Intralaboratory reproducibility was evaluated using three independent experimental replicates per strain under both substrate loads. For the 2 mg milk condition, coefficients of variation (CV) ranged from 7.72% to 15.35%. PA14, PAO1, and DVL1758 exhibited high precision (CV < 10%), whereas the LB control, PAO1Δ*lasR*Δ*rhlR*, and ATCC 9027 showed moderate variability (CV ≈ 11.63–15.35%) (Table 2). Under the 5 mg condition, overall precision remained acceptable (CV = 4.99%–18.07%): the LB control and PAO1 displayed the lowest dispersion (CV = 4.99% and 8.12%), while PAO1Δ*lasR*Δ*rhlR* and ATCC 9027 exhibited higher variability (up to 18.07%), consistent with a low-activity regime in which small absolute differences yield larger relative variation.

Beyond precision, Table 2 summarizes the assay’s capacity to resolve strain-dependent protease-associated phenotypes. At 2 mg, the readout separated two statistically distinct groups: a high-turbidity/low-degradation phenotype (LB control, PAO1Δ*lasR*Δ*rhlR*, ATCC 9027; superscript b) and a low-turbidity/high-degradation phenotype (PAO1, PA14, DVL1758; superscript a). At 5 mg, the same rank order was preserved, with improved stratification into high (c), intermediate (b), and low (a) turbidity phenotypes. Collectively, CV values remained below 20% across strains and conditions, supporting that liquid-phase milk turbidimetry is sufficiently reproducible for comparative protease phenotyping and screening, while remaining robust across substrate loads.

To evaluate whether the modest differences in endpoint culture density influenced the interpretation of protease activity, residual turbidity values were normalized to the corresponding mean OD600 for each strain (Table 3).

**TABLE 3:** Sensitivity analysis of residual turbidity normalized to endpoint culture density (A600/OD600)^*a*^^,^^*b*^

Strain	Endpoint OD600	Mean A600 (2 mg)	A600/OD600 (2 mg)	Mean A600 (5 mg)	A600/OD600 (5 mg)
PAO1	3.84	0.24	0.062^b^	0.67	0.174^c^
PA14	3.40	0.28	0.082^c^	0.71	0.208^c^
DVL1758	3.59	0.31	0.086^a^	0.69	0.192^c^
ATCC 9027	4.00	0.38	0.095^a^	1.19	0.297^b^
PAO1Δ*lasRΔrhlR*	3.69	0.45	0.121^a^	1.64	0.444^a^

^*a*^ Data were normalized using the corresponding mean endpoint OD600 for each strain. The analysis is presented as a sensitivity assessment to evaluate whether modest differences in bacterial growth affected the interpretation of extracellular protease activity.

^*b*^ Different letters indicate significant differences (one-way ANOVA + Tukey’s HSD, α = 0.05); lower A600 indicates higher protease activity.

Normalization did not alter the relative ranking of strains or the discrimination between low- and high-protease phenotypes, supporting the conclusion that the observed differences in milk turbidity primarily reflect variation in extracellular proteolytic activity rather than differences in bacterial growth.

### Robustness across substrate loads

**Fig 4:**
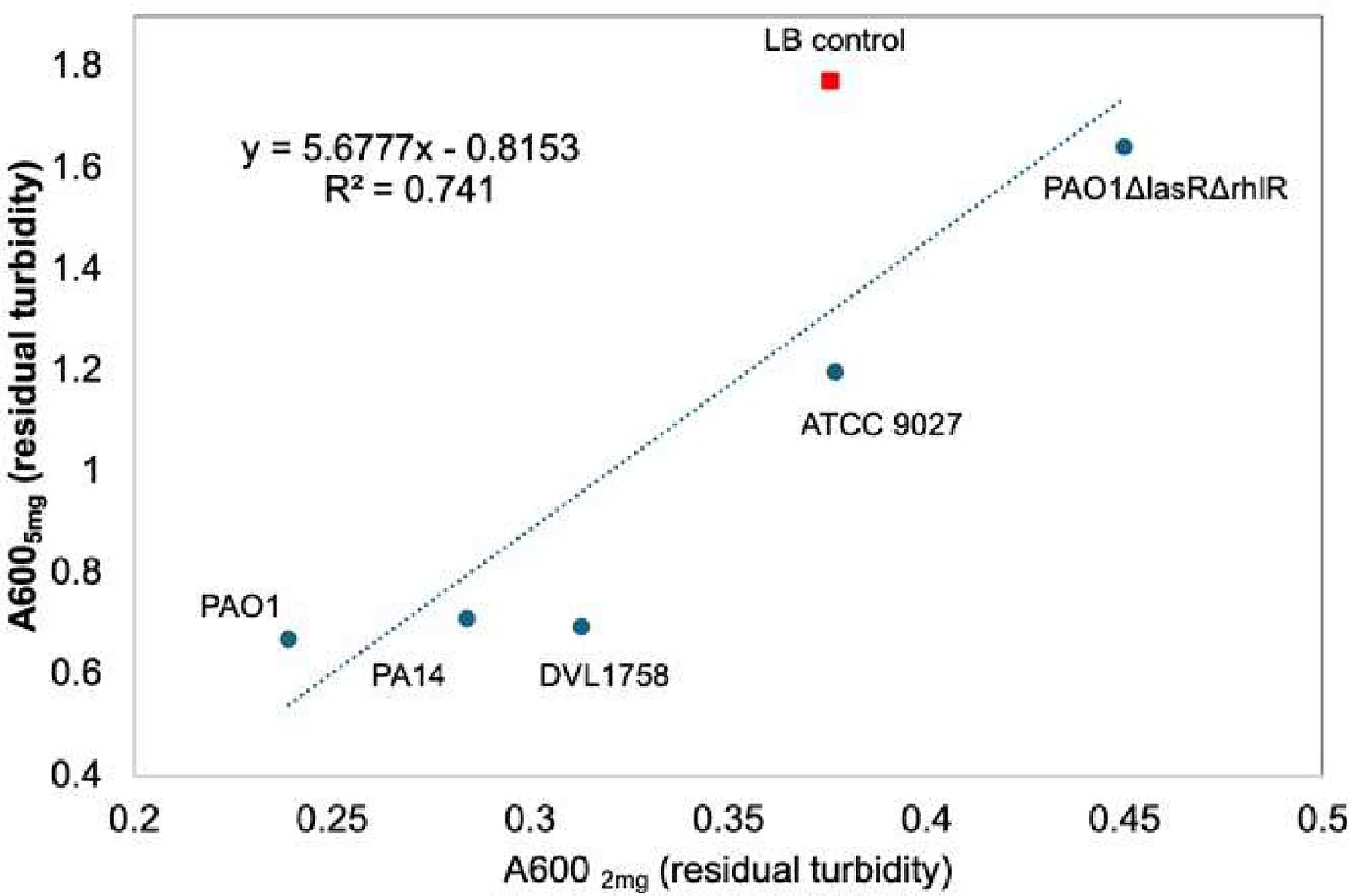
Internal consistency of milk turbidimetry across substrate loads (2 mg vs 5 mg milk per reaction). Mean A600 values obtained under both substrate-load conditions were compared across strains (mean ± SD of three independent biological replicates, each measured in triplicate technical replicates; *n* = 3 biological replicates per strain), including the LB negative control (red square). A strong positive correlation was observed (Pearson *r* = 0.861, *P* = 0.0277, *n* = 6), with a linear fit of A600₅mg = −0.815 + 5.678 × A600₂mg (*R*² = 0.741). Excluding the LB negative control further strengthened the association among biological samples (*r* = 0.954, *P* = 0.0118, *n* = 5; linear fit: A600₅mg = −0.667 + 4.959 × A600₂mg; *R*² = 0.910).

Figure 4 supports assay robustness by showing that strain ranking was preserved across substrate loads. Mean A600 values obtained under the 2 mg and 5 mg conditions were strongly correlated (Pearson *r* = 0.861, *P* = 0.0277; *n* = 6), indicating that strains classified as low- vs high-protease phenotypes at 2 mg retained the same relative ranking at 5 mg. The accompanying linear regression is presented as a descriptive assessment of concordance between substrate-load conditions rather than as a predictive model.

Excluding the LB medium control (red square) further strengthened the association (*r* = 0.954; *R*² = 0.910; *n* = 5), consistent with interpreting robustness chiefly across biological samples, whereas the medium-only control serves as a non-biological reference. Together, these results demonstrate internally consistent quantitative readouts across a practical range of milk loads and support the assay’s suitability for comparative total protease phenotyping and screening, while allowing flexible substrate selection depending on whether discrimination (lower load) or baseline signal (higher load) is prioritized.

Following the cross-load correlation analysis (Fig. 4), the key performance characteristics of the assay are consolidated in Table 4 to provide a practical summary of its analytical behavior and recommended operating modes. Consistent with the strong 2 mg–5 mg association, the readout (residual turbidity at A600) yields internally consistent rankings across loads, while maintaining acceptable intralaboratory precision, with coefficients of variation generally below 20%, a commonly used benchmark for biological assay reproducibility and bioanalytical method validation (25, 26). Importantly, Table 4 highlights the complementary strengths of each operating mode: the 2 mg load maximizes phenotypic discrimination among strains, whereas the 5 mg load provides a higher baseline turbidity that can be advantageous when proteolytic activity is weak or near the lower end of the dynamic range. Together with the preservation of strain rank order shown in Fig. 4, this summary supports the use of milk turbidimetry as a flexible, even microplate-compatible platform for comparative protease phenotyping across varying signal-range needs.

**TABLE 4:** Assay performance summary and recommended operating conditions^*a*^

Performance element	Metric reported	Milk load (2 mg)	Milk load (5 mg)	Practical interpretation
Readout	Residual turbidity (A600)	√	√	Lower A600 indicates higher milk clearance (greater total proteolysis)
Replication	Biological replicates per strain	*n* = 3	*n* = 3	Supports estimation of SD/CV and group separation
Precision	CV range (%)	7.72–15.35	4.99–18.07	CV <20% indicates acceptable intralaboratory reproducibility
Discrimination	Strain effect	Significant (ANOVA, *P* < 0.001)	Significant (ANOVA, *P* < 0.001)	Resolves distinct protease-associated phenotypes (Tukey HSD)
Robustness across loads	Pearson correlation (2 mg vs 5 mg)	*r* = 0.861 (*P* = 0.0277), *R*² = 0.741	–	Preserved ranking across loads supports internal consistency
Robustness excluding LB	Pearson correlation (excluding LB control)	*r* = 0.954 (*P* = 0.0118), *R*² = 0.910	–	Association strengthens when comparing strains only
Recommended use	Operating mode	Higher phenotypic resolution	Higher baseline signal	2 mg for discrimination; 5 mg for robustness when activity is weak

^*a*^ A600 values are reported as the mean ± SD (*n*=3 independent biological replicates per strain), with each biological replicate measured in triplicate technical replicates. CV (%) was calculated as (SD/mean) × 100. Group letters indicate significant differences determined by one-way ANOVA followed by Tukey’s HSD test (*α* = 0.05) within each milk-load condition. “–” indicates that the corresponding analysis was not applicable to that condition. “√ ” indicates that the performance parameter was evaluated under the indicated milk load condition (2 or 5 mg).

### Comparative benchmarking of total protease readouts and practical trade-offs

Table 5 benchmarks liquid-phase milk turbidimetry against commonly used *P. aeruginosa* protease readouts by summarizing substrate type, workflow complexity, signal directionality, throughput, and practical limitations. Existing approaches span (i) complex-substrate proxies for global proteolysis (azocasein) (27), (ii) more selective assays biased toward specific protease classes or substrates (elastin–Congo red [28], fluorogenic peptides), and (iii) orthogonal validation tools (zymography, OPA/TNBS chemistry) (29).

**TABLE 5:** Benchmarking of protease readouts relative to liquid-phase milk turbidimetry^*a*^

Method	Substrate	Readout	Best captures	Major limitation	Typical use	Reference
Liquid-phase milk turbidimetry	Skim milk proteins	A600 (↓ = more proteolysis)	Global secreted proteolysis proxy	Not protease-specific; substrate variability (brand/lot)	High-throughput phenotyping	This study
Skim-milk agar clearing	Milk in agar	Halo size	Global proxy +growth/diffusion	Semi-quantitative; diffusion confounding	Rapid screening	(30)
Azocasein assay	Azocasein	A440/A450 (↑)	Global proteolysis proxy	More handling steps (stop/clarify)	Quantitative validation	(28)
Elastin–Congo red	Elastin-based	A495 (↑)	Elastase-like activity bias	Specialized substrate; not “total protease”	Targeted elastase readout	(27)
Fluorogenic peptides	Defined peptides	Fluorescence (↑)	Substrate-specific kinetics	Cost + substrate bias	Mechanistic/inhibitor studies	(17)
Zymography	Gelatin/casein gels	Activity bands	Activity “fingerprint”	Low throughput; semi-quantitative	Orthogonal validation	(31)

^*a*^ Symbols: “↓” indicates that increased proteolytic activity results in a lower measured signal (e.g., decreased A600 due to milk degradation in the liquid-phase turbidimetry assay). “↑” indicates that increased proteolytic activity results in a higher measured signal (e.g., increased absorbance or fluorescence due to substrate cleavage).

As a global proteolysis proxy, milk turbidimetry provides a low-barrier, microplate-compatible workflow using widely available skim milk and a simple Tris–CaCl₂ buffer, enabling rapid comparative phenotyping without specialized substrates. Relative to skim-milk agar clearing, the liquid A600 readout is more directly quantifiable and less affected by diffusion-based halo artifacts. Relative to azocasein, it reduces hands-on steps by avoiding stop/precipitation/clarification reactions (e.g., TCA precipitation and downstream handling), thereby improving scalability, at the cost of being an indirect optical proxy that benefits from clarified supernatants and consistent processing.

More selective assays (elastin–Congo red and fluorogenic peptides) offer higher biochemical specificity and, in some cases, sensitivity but require specialized substrates and their signals reflect the chosen target (often elastase-like activity or peptide-sequence dependence) rather than total proteolysis. Orthogonal approaches such as zymography and OPA/TNBS provide complementary validation (activity banding or chemical quantification of proteolysis products) but are lower-throughput and/or more reagent-intensive (29). Taken together, Table 4 positions milk turbidimetry as a cost-effective, accessible method for high-throughput comparative profiling of secreted protease phenotypes, while clarifying scenarios where target-specific or orthogonal essays are preferable.

To complement this benchmarking overview, Table 6 provides a methodological comparison of liquid-phase milk turbidimetry and representative conventional protease assays, highlighting differences in substrate composition, buffer requirements, reaction setup, incubation conditions, signal generation, and practical applications. This comparison further illustrates the balance between analytical simplicity, throughput, specificity, and operational complexity among commonly used approaches for assessing proteolytic activity.

**TABLE 6:** Methodological comparison of protease readouts: liquid-phase milk turbidimetry versus conventional assays

Assay format (example)	Substrate	Buffer/cofactors	Sample substrate setup	Incubation	Readout	Signal direction	Advantage/limitation
This study: liquid-phase milk turbidimetry (Condition A)	Commercial skim milk powder (2%, wt/vol)	0.1 M Tris-HCl pH 7.0 + 1 mM CaCl₂	100 µL supernatant + 100 µL milk substrate + 800 µL buffer (1 mL total)	37°C, shaking (220 rpm), 3 h	A600 (turbidity)	Lower A600 = higher proteolysis	Higher phenotypic resolution for strain discrimination
This study: liquid-phase milk turbidimetry (Condition B)	Commercial skim milk powder (2%, wt/vol)	0.1 M Tris-HCl pH 7.0 + 1 mM CaCl₂	100 µL supernatant + 250 µL milk substrate + 650 µL buffer (1 mL total)	37°C, shaking (220 rpm), 3 h	A600 (turbidity)	Lower A600 = higher proteolysis	Greater signal stability; may reduce fine discrimination vs Condition A
Elastin–Congo red (ECR) elastase assay (literature example; protocol varies by study)	Elastin–Congo red (ECR), typically 5 mg	Tris buffer (often pH 7.5– 8.0) with Ca²^+^ (e.g., 0.5– 1 mM)	Supernatant + buffer + ECR; volumes vary (commonly 1 mL total)	Typically 37°C with shaking; 3–24 h depending on study	A495 (released dye)	Higher A495 = higher elastolysis	Often used as an elastase-like readout (LasB-associated depending on conditions)
Skim-milk agar plate clearing assay (literature; qualitative/semi- quantitative)	Skim milk in agar (e.g., 1%–2%, wt/vol)	Growth medium agar (e.g., LB agar)	Spot or well inoculation; zone-of-clearing measured	Typically 30–37°C; 16–24 h	Clearing halo diameter/area	Larger halo = higher proteolysis	Diffusion and growth confound strict quantification; useful for rapid screening
Azocasein/ chromogenic casein assay (literature; common workflow)	Azocasein (or chromogenic casein)	Often phosphate or Tris buffer near neutral pH; may include Ca²^+^	Supernatant + azocasein; stop with TCA; clarify; alkaline development	Typically 25–37°C; 30–60 min	A440 (soluble dye fragments)	Higher A440 = higher proteolysis	Quantitative but involves extra handling steps (TCA precipitation/centrifugation)
Fluorogenic peptide substrate assay (literature; substrate-specific)	Fluorogenic peptides (various)	Assay-specific buffer	Microplate format; reaction volumes typically 50–200 µL	Typically 25–37°C; kinetic readout possible	Fluorescence (Ex/Em substrate-dependent)	Higher fluorescence = higher activity	High sensitivity; cost and equipment requirements are higher; specificity depends on peptide
Gelatin/casein zymography (literature; orthogonal validation)	Gelatin or casein copolymerized in SDS-PAGE gel	Renaturation/development buffers	Electrophoresis + renaturation + incubation; visualization by staining	Varies (hours to overnight)	Band clearing intensity	Stronger clearing = higher activity	Provides activity band patterns; low throughput and semi-quantitative
OPA/TNBS (free amino groups) assays (literature; orthogonal chemistry)	Protein substrate + derivatization reagent (OPA or TNBS)	Assay-specific reagents/buffers	Measure soluble peptides/amino groups after proteolysis	Varies	Absorbance (reagent-dependent)	Higher absorbance = more amino groups	Useful orthogonal validation; adds reagents and potential matrix interferences

### Cost and accessibility benchmarking of protease phenotyping assays

To complement the methodological comparison (Table 6), Table 7 summarizes practical considerations that often determine assay choice in routine phenotyping, including reagent availability, relative consumable cost, and workflow complexity.

**TABLE 7:** Practical benchmarking of protease assays by scalability and resource requirements

Method	Equipment	Hands-on complexity	Throughput	Cost per sample (relative)	Note
Liquid-phase milk turbidimetry	Plate reader (A600)	Low	High	Very low	Best for routine screening/phenotyping
Skim-milk agar clearing	Incubator	Low–moderate	Medium	Very low	Diffusion/growth confound quantification
Azocasein assay	Reader + often centrifuge	Moderate–high	Medium	Medium	More steps; timing sensitive
Elastin–Congo red	Reader + shaker	Moderate	Medium–low	Medium–high	Elastase-biased; substrate limiting
Fluorogenic peptides	Fluorescence reader	Moderate	High	High	High sensitivity/kinetics
Zymography	Gel system + imaging	High	Low	Medium	Great fingerprints; not scalable

From an implementation standpoint, liquid-phase milk turbidimetry is advantageous because it relies on inexpensive, widely available inputs (skim milk powder and Tris–CaCl₂ buffer) and a standard absorbance microplate reader. This combination minimizes per-sample consumable cost and reduces hands-on steps, making the workflow practical for routine use and for screening panels beyond a small number of strains under standardized conditions.

To provide a practical cost comparison between substrate-based assays, the approximate per-reaction cost was estimated using representative commercial prices. Elastin–Congo red substrate (Sigma-Aldrich) is typically supplied at approximately $400 USD per 5 g, corresponding to ~0.40 USD per reaction when 5 mg substrate is used. In contrast, skim milk powder used in this study (commercial Svelty formulation) costs approximately $23 USD per 1.6 kg, corresponding to ~0.00007 USD per reaction at the same 5 mg substrate load. Thus, the milk-based assay reduces substrate cost by approximately four orders of magnitude (~10³–10⁴-fold) relative to elastin–Congo red, highlighting a major advantage for large-scale phenotyping and routine laboratory implementation.

By comparison, elastin–Congo red assays, commonly used as elastase-like readouts, typically require specialized substrates and may show batch-to-batch variability, increase cost and limiting routine adoption. Azocasein provides a broadly responsive proteolysis measure but often involves additional handling steps (for example, stop/precipitation and clarification, followed by signal development), increasing labor, consumables, and timing sensitivity. Skim-milk agar clearing remains attractive for low-cost preliminary screening, but diffusion and growth effects complicate strict quantification and cross-study comparability. Fluorogenic peptide assays enable sensitive, kinetic measurements, yet are more expensive and require fluorescence-capable instrumentation; moreover, signals depend on the chosen substrate sequence and may not represent total proteolysis. Finally, zymography and OPA/TNBS-type assays can serve as orthogonal validation tools (activity banding or chemical quantification of proteolysis products) but are more labor-intensive and generally less suited to routine screening.

Table 5 highlights that assay selection involves trade-offs among cost, accessibility, specificity, and operational complexity. Within this landscape, liquid-phase milk turbidimetry is positioned as a low-cost and highly accessible option for comparative profiling of secreted proteolytic phenotypes, with targeted substrates or orthogonal methods best reserved for specificity-driven or validation-focused applications.

## DISCUSSION

In this study, we developed and validated a liquid-phase milk turbidimetry-based spectrophotometric assay to detect total secreted protease activity in liquid cultures of *P. aeruginosa*. Our results demonstrate that this method reliably discriminates among strains with different proteolytic phenotypes and provides reproducible measurements with inexpensive, readily accessible reagents. To our knowledge, this study provides a standardized and experimentally validated liquid-phase milk turbidimetry workflow for comparative analysis of total secreted protease activity in *P. aeruginosa*.

The markedly reduced proteolytic activity observed in the PAO1Δ*lasR*Δ*rhlR* mutant confirms the central role of QS systems in regulating extracellular protease production, previously reported (13, 23). Previous studies have shown that LasR and RhlR control the expression of major protease-encoding genes, including *lasB* and *aprA*, and our findings are consistent with these regulatory mechanisms (32). In contrast, the reference strains PAO1 and PA14 exhibited the highest levels of milk degradation, reflecting their well-documented capacity to secrete multiple virulence-associated proteases (9, 33).

The environmental isolate DVL1758 displayed an intermediate proteolytic phenotype, highlighting the ability of the assay to detect gradual variations in enzyme production among different isolates. Similarly, the low activity observed in ATCC 9027 supports previous reports describing its reduced protease secretion (34, 35). Together, these results indicate that the assay showed biologically meaningful differences in secreted protease activity across genetically and ecologically diverse strains.

Reproducibility analysis revealed acceptable coefficients of variation, particularly in strains with high proteolytic activity, demonstrating good intra-assay precision. The slightly higher variability observed in strains with low proteolytic activity is likely attributable to the limited change in milk turbidity produced by these samples. Under such conditions, small experimental fluctuations, including pipetting variation, minor differences in substrate dispersion, and instrumental noise, represent a larger proportion of the measured signal, resulting in higher coefficients of variation. This behavior is a common characteristic of turbidity-based assays operating near the lower limit of their analytical response rather than an indication of poor assay performance. Importantly, despite this increased relative variability, all CV values remained below 20%, and the assay consistently distinguished low- and high-protease phenotypes, supporting its robustness for comparative analyses.

The comparison of substrate concentrations further indicated that lower milk amounts enhanced analytical sensitivity, whereas higher concentrations improved signal robustness. This flexibility allows users to adjust assay conditions according to specific experimental objectives, such as high-resolution phenotypic discrimination or routine screening.

In addition to plate-based milk or gelatin assays, several methods have traditionally been used to evaluate proteolytic activity in *P. aeruginosa* (12), including azocasein degradation assays, chromogenic substrates, and fluorogenic peptide-based systems. Although plate assays enable rapid screening, they provide only semi-quantitative information and are influenced by diffusion effects and colony morphology. Azocasein-based methods offer quantitative measurements but require multiple processing steps that limit throughput and increase variability.

Chromogenic and fluorometric assays based on synthetic peptide substrates provide high sensitivity and specificity toward individual proteases, such as LasB or LasA. However, these approaches require specialized equipment, costly reagents, and prior knowledge of substrate specificity. Moreover, they typically focus on single enzymes and, therefore, do not reflect the combined activity of the diverse protease repertoire secreted by *P. aeruginosa* (12, 17).

In contrast, the milk-based assay described here provides an integrated measurement of extracellular proteolytic activity in liquid cultures. Using a complex protein substrate, the assay reflects the combined activity of multiple secreted proteases present in the culture supernatant. Rather than replacing substrate-specific assays, it provides a complementary phenotypic readout that is well suited for comparative analyses of overall secreted protease activity. In addition, minimal sample processing reduces technical variability and facilitates adaptation to medium-throughput screening formats.

A major advantage of the proposed method is its simplicity and low cost, as it requires no specialized instrumentation beyond a basic spectrophotometer or microplate reader. The use of readily available reagents enables implementation in laboratories with limited resources and supports broad accessibility to quantitative protease analysis. Several limitations should nevertheless be considered. First, the assay provides an integrated measure of extracellular proteolytic activity and therefore does not permit discrimination among the contributions of individual proteases, such as LasB, AprA, LasA, PIV, or other secreted enzymes. Consequently, the assay cannot replace targeted biochemical, genetic, or molecular approaches when mechanistic characterization of specific proteases is required. Future studies combining this assay with protease-deficient mutants, selective inhibitors, or purified enzymes will be valuable for defining the relative contribution of individual extracellular proteases to the overall milk degradation signal. Second, substrate composition, pH, medium components, and endogenous inhibitors may influence substrate degradation and should be carefully controlled. Because the skim milk substrate was prepared in water, the final Tris-HCl and CaCl₂ concentrations differed slightly between substrate-load conditions. However, these concentrations remained within ranges commonly used for extracellular protease assays, and the strong correlation and preservation of strain ranking between conditions suggest that substrate load was the primary factor influencing assay performance (12). Third, the culture supernatants were clarified by centrifugation but were not subjected to 0.22 μm filtration before the assay. Although residual bacterial carryover was not directly evaluated, all strains underwent identical sample preparation, and the QS-deficient PAO1Δ*lasR*Δ*rhlR* mutant consistently exhibited minimal milk degradation under both assay conditions. These observations suggest that any potential carryover did not materially affect the comparative strain rankings obtained in this study.

Finally, two additional points must be considered. First, autoclaving may modify the structural properties of milk proteins through heat-induced denaturation and changes in casein-associated organization. Although such treatment is routinely used in skim-milk-based protease assays and was applied uniformly throughout this study, differences in substrate preparation protocols could influence absolute signal intensity and should, therefore, be standardized when comparing results across laboratories. Second, commercial skim milk powders may differ in protein composition and additives depending on the manufacturer. Such differences could influence absolute turbidity values and assay sensitivity. Although a single commercial product was used consistently throughout this study to minimize variability, users should standardize the milk source within a study, and future work should evaluate assay performance across different commercial formulations.

Furthermore, the assay was validated using a limited panel of laboratory, environmental, and clinical strains selected to represent contrasting proteolytic phenotypes. Although these strains encompass distinct regulatory and phenotypic backgrounds, additional validation using larger collections of clinical and environmental isolates, naturally occurring QS-deficient strains, hyperproteolytic variants, and other regulatory mutants would further establish the general applicability of the method. Inter-laboratory reproducibility was also not evaluated and represents another important aspect for future validation.

Despite these limitations, the assay is well-suited for applications including quorum-sensing studies, screening of regulatory mutants, identification of protease inhibitors, and comparative analyses of clinical, environmental, and laboratory strains. Because the assay relies on a simple absorbance measurement at 600 nm, it could be readily adapted to microplate readers for medium-throughput applications. However, this implementation was not experimentally evaluated in the present study and should be validated in future work.

Overall, this work provides an accessible, reliable, and practical tool for evaluating secreted protease activity in *P. aeruginosa*. By combining simplicity, reproducibility, and biological relevance, the proposed assay complements existing methodologies and expands the experimental toolkit available for studying the regulation and functional roles of bacterial extracellular proteases.

### Conclusions

This work establishes a liquid-phase skim-milk turbidimetric assay to quantify total secreted protease activity in *Pseudomonas aeruginosa* by measuring turbidity loss at 600 nm (A600). Because all strains reached comparable endpoint densities (OD600 without significant differences), the strain-dependent differences in residual turbidity are best interpreted as true variation in extracellular proteolysis rather than biomass effects. The assay reproducibly separated low-, intermediate-, and high-degradation phenotypes at both 2 mg and 5 mg substrate loads, with patterns consistent with QS control (the PAO1Δ*lasR*Δ*rhlR* mutant showed minimal milk clearance relative to high-activity reference strains). Intralaboratory reproducibility was acceptable for a biological assay (CV < 20% across strains and conditions), and robustness across loads was supported by the strong correlation and linear relationship between mean A600 values at 2 mg and 5 mg, preserving strain ranking even when the medium-only control was excluded. Practically, 2 mg maximizes phenotypic discrimination, whereas 5 mg provides a higher baseline signal when activity is weak. Benchmarking against established readouts positions milk turbidimetry as a low-cost, widely accessible option for comparative protease phenotyping and screening, while noting that specialized substrates or orthogonal assays remain preferable when higher specificity or mechanistic validation is required.

Future work should focus on extending the assay to larger isolate panels and strengthening cross-laboratory standardization using shared reference strains and minimal QC criteria (e.g., substrate lot checks and clarification requirements).

## Supplementary Material

Reviewer comments

## SUPPLEMENTAL MATERIAL

OPEN PEER REVIEW reviewer-comments.pdf An accounting of the reviewer comments and feedback.

ASM does not own the copyrights to Supplemental Material that may be linked to, or accessed through, an article. The authors have granted ASM a non-exclusive, world-wide license to publish the Supplemental Material files. Please contact the corresponding author directly for reuse.
