# Supplementary material for: Liquid-phase milk turbidimetry enables rapid phenotyping of secreted protease activity in Pseudomonas aeruginosa at less than one cent per sample
Source: Microbiol Spectr. 2026 Sep 10;14(10):e01271-26. doi: 10.1128/spectrum.01271-26 (PMC13689426; doi:10.1128/spectrum.01271-26)
Supplement: Supplementary file 1 [file reviewer-comments.pdf]

ASM | Microbiology Spectrum

# Liquid-phase milk turbidimetry enables rapid phenotyping of secreted protease activity in *Pseudomonas aeruginosa* at less than one cent per sample

Selene García-Reyes, Karla Enríquez-Ríos, and Juan Pineda Licona

*Corresponding Author(s): Selene García-Reyes, Universidad Nacional Autonoma de Mexico*



| **Review Timeline:** | Submission Date: | April 13, 2026 |
|---|---|---|
| | Editorial Decision: | June 15, 2026 |
| | Revision Received: | July 29, 2026 |
| | Accepted: | August 10, 2026 |

*Editor: Sadjia Bekal*

*Reviewer(s): Disclosure of reviewer identity is with reference to reviewer comments included in decision letter(s). The following individuals involved in review of your submission have agreed to reveal their identity: Yinghua Xiao (Reviewer #1); Dina A Moustafa (Reviewer #2)*

## Transaction Report:

(Note: With the exception of the correction of typographical or spelling errors that could be a source of ambiguity, letters and reports are not edited. The original formatting of letters and referee reports may not be reflected in this compilation.)

**DOI: https://doi.org/10.1128/spectrum.01271-26**

Re: Spectrum01271-26 (**Liquid-phase milk turbidimetry enables rapid phenotyping of secreted protease activity in *Pseudomonas aeruginosa* at less than one cent per sample**)

Dear Dr. Selene García Reyes:

Thank you for the privilege of reviewing your work. Below you will find my comments, instructions from the Spectrum editorial office, and the reviewer comments.

Please return the manuscript within 60 days; if you cannot complete the modification within this time period, please contact me. If you do not wish to modify the manuscript and prefer to submit it to another journal, notify me immediately so that the manuscript may be formally withdrawn from consideration by Spectrum.

**Revision Guidelines**
To submit your modified manuscript, log into the submission site at https://spectrum.msubmit.net/cgi-bin/main.plex. Go to Author Tasks and click the appropriate manuscript title to begin. The information you entered when you first submitted the paper will be displayed; update this as necessary. Note the following requirements:

• Upload point-by-point responses to the issues raised by the reviewers in a file named "Response to Reviewers," NOT in your cover letter.
• Upload a compare copy of the manuscript (without figures) as a "Marked-Up Manuscript" file.
• Upload a clean .DOC/.DOCX version of the revised manuscript and remove the previous version.
• Each figure must be uploaded as a separate, editable, high-resolution file (TIFF or EPS preferred), and any multipanel figures must be assembled into one file.
• Any supplemental material intended for posting by ASM should be uploaded with their legends separate from the main manuscript. You can combine all supplemental material into one file (preferred) or split it into a maximum of 10 files with all associated legends included.

For complete guidelines on revision requirements, see our Submission and Review Process webpage. Submission of a paper that does not conform to guidelines may delay acceptance of your manuscript.

**Data availability:** ASM policy requires that data be available to the public upon online posting of the article, so please verify all links to sequence records, if present, and make sure that each number retrieves the full record of the data. If a new accession number is not linked or a link is broken, provide Spectrum production staff with the correct URL for the record. If the accession numbers for new data are not publicly accessible before the expected online posting of the article, publication may be delayed; please contact production staff (Spectrum@asmusa.org) immediately with the expected release date.

**Publication Fees:** For information on publication fees and which article types are subject to charges, visit our website. If your manuscript is accepted for publication and any fees apply, you will be contacted separately about payment during the production process; please follow the instructions in that e-mail. Arrangements for payment must be made before your article is published.

**ASM Membership:** Corresponding authors may join or renew ASM membership to obtain discounts on publication fees. Need to upgrade your membership level? Please contact Customer Service at Service@asmusa.org.

The ASM Journals program strives for constant improvement in our submission and publication process. Please tell us how we can improve your experience by taking this quick Author Survey.

Thank you for submitting your paper to Spectrum.

Sincerely,
Sadjia Bekal
Editor
Microbiology Spectrum

Reviewer #1 (Comments for the Author):

This manuscript presents a low-cost, liquid-phase skim-milk turbidimetric assay for rapid phenotyping of secreted protease activity in Pseudomonas aeruginosa. The study is relevant and potentially useful because it offers a simple and accessible workflow for comparative analysis of extracellular proteolysis across strains with different quorum-sensing backgrounds and proteolytic phenotypes.

General comments: The paper is generally well organized and includes useful comparative data across strains and substrate loads. However, before publication, I recommend substantial revision to improve readability, reduce repetition, strengthen methodological clarity, and tighten the interpretation of results.

The manuscript presents milk proteins, mainly caseins, as a complex but widely available substrate, while also arguing for improved standardization and higher-throughput comparison. The authors should explain more clearly how the use of a complex natural substrate aligns with the goal of assay standardization, and whether this introduces variability that may limit comparability across studies or laboratories. A related concern is the use of a commercial skim milk powder product that includes collagen, while the full ingredient composition is not reported. The authors should clarify all relevant ingredients in the product and discuss whether collagen or other additives could influence assay performance, reproducibility, or transferability when other researchers use different skim milk powders or formulations.

Another general concern relates to the use of OD600 as the sole measure of bacterial concentration or culture density in the Methods and Results sections. Optical density is only an indirect proxy and may be influenced by strain-dependent differences in cell morphology, aggregation, or extracellular polymeric substance production. The authors should justify why direct enumeration methods, such as plate counting, were not used to support comparability across strains, particularly because the assay interpretation depends on excluding biomass-related effects.

Specific comments:

Line 43: Please remove the period between "biology" and reference (9).

Line 48: The sentence ending with "inference" appears to lack an appropriate supporting reference. Please add the relevant citation.

Line 51: The first part of the sentence be rephrased to: The production of these proteases in...".

Line 93: Please add "See Table 1" following "ATCC 9027".

Line 113: The skim milk solution and assay buffer were autoclaved at 121 {degree sign}C for 15 min. Have the authors considered whether this treatment could alter the milk proteins and affect assay performance? Please also discuss whether heat-induced changes in skim milk components, such as casein-related structure or other protein denaturation, could influence reproducibility or comparability of the assay.

Lines 124-127: In Conditions A and B, different volumes of milk substrate and assay buffer are used. As a result, the final concentrations of assay components, including Tris and $CaCl_2$, may differ between the two conditions. Please clarify whether this could introduce bias into the comparison, particularly if the higher $CaCl_2$ concentration in Condition B may influence protease activity or assay readout independently of the milk load.

Table 2: The turbidity values (A600) for both the 2 mg and 5 mg conditions are already presented in Figures 2 and 3. As a result, Table 2 partly repeats information that is already shown elsewhere, with CV% being the main additional value. The authors may restructure the presentation to reduce redundancy, for example by simplifying the table, integrating only the variability data, or moving part of the material to the Supplementary Information.

Figures 2 and 3: The authors should explain more clearly the behavior of strain DVL1758 across the two milk substrate loads. In the 2 mg condition (Figure 2), DVL1758 appears not to differ clearly from the LB control, the mutant, and ATCC 9027, whereas in the 5 mg condition (Figure 3) it is grouped together with PAO1 and PA14 and separated from the low-activity group. This shift in statistical grouping is important for interpretation and deserves clearer explanation in the Results or Discussion.

Lines 196 and 226: Please double-check the table citations in the Results section. It is not clear whether the manuscript should refer to Table 1 (strain information) in these two lines.

Figure 4: In the figure legend, the authors state that the association becomes stronger when the LB control is excluded. However, the figure itself appears to show only the analysis including the LB control. It would be more reasonable to present both versions, with and without the LB control.

Line 310: The manuscript states that CV < 20% indicates acceptable precision. Please clarify the basis for this criterion and provide an appropriate reference to support it.

Table 4, Readout column: Please explain the meaning of the symbols used in the Readout column, such as the up arrow, down arrow, and equals sign. Their interpretation is not currently defined, which may make the table unclear to readers.

Table 4, References column: For the skim-milk agar clearing method, the reference is given as "this study." If this technique is already established and only discussed or used here for comparison, the authors should cite the original article(s) describing or applying this method.

Lines 365 and 401: Please check the consistency of table numbering throughout the manuscript. Table 6 appears to be mentioned earlier in the text, whereas the first mention of Table 5 occurs later. The order of table citations should be made fully consistent.

Reviewer #2 (Comments for the Author):

The manuscript by EnrÃquez-RÃos et al â€œLiquid-phase milk turbidimetry enables rapid phenotyping of secreted protease activity in Pseudomonas aeruginosa at less than one cent per sampleâ€ introduces a cost-effective, liquid-phase skim milk turbidimetry assay designed to quantify total secreted protease activity in Pseudomonas aeruginosa. The manuscript offers a practical low coast tool using straightforward, accessible, and reproducible protocol. The text is well-structured, clearly articulated, and fills a notable methodological gap. However, to maximize the manuscript's clarity and impact, several key areas require attention

Major comments:

-The authors state "A600 differences were interpreted without additional OD normalization, given that endpoint OD600 was statistically indistinguishable across strains." OD600 ≈ 3.4-4.0 represents nearly 20% variation in biomass and such small difference in culture density can influence secreted enzymes and supernatant activity. A more rigorous approach should be included (at least a sensitivity analysis showing that normalization does not change strain ranking)

-Can the authors clarify why low-activity strains show higher variability.

-The author's claim of the broad applicability of the assay, this is not fully supported with only five strains are tested. While the panel includes reference strains and one QS mutant, the manuscript claims broad applicability ("diverse strain backgrounds"). Additional isolates, such as clinical isolates known with loss-of-function mutations in QS and/or QS mutants beyond lasR/rhlR, or hyperproteolytic variants would strengthen the generalizability of the assay.

-The assay uses a commercial milk powder with collagen additives; the authors need to justify the choice of this specific milk product and include a cautionary note in the method section. Other milk brands and lots should be tested to address the milk composition and how it varies across brands and lots.

-Have the authors taken into consideration the collagen additives and the possibility of these peptides interfering with turbidity or protease susceptibility?

-What is the rationale of autoclaving milk? This process can denature proteins in inconsistent ways.

-The manuscript does not validate which proteases contribute most to the signal.
For example, LasB, AprA, LasA, and PIV have different substrate preferences, it is difficult to interpret the biological meaning of the A600 readout. Did the authors consider using specific inhibitor studies and/or knockout panels. The manuscript would benefit from at least one validation experiment.

-The manuscript reports means and SDs from n = 3 biological replicates but no technical replicates are mentioned. Given the small sample size, the authors should justify the statistical approach or provide more sample size

-The authors are emphasizing the intralaboratory reproducibility, yet all data are reported from a single laboratory. For a method paper, cross-lab validation is highly desirable. Even a small two-lab comparison would significantly increase confidence in the assay's robustness.

.In the discussion section, the comparison to elastin-Congo red and azocasein assays is strong, but unless sufficient evidence are provided, the authors should avoid implying that milk turbidity is inherently more "biologically relevant"..

Minor comments:

Line 11-13: This sentence is correct but overly long. Consider splitting for clarity: "Secreted proteases are major virulence determinants in Pseudomonas aeruginosa and vary widely across strains and quorum-sensing (QS) backgrounds, thereby motivating the development of simple, standardized phenotyping tools."

The Introduction is overly long and repeat background information on QS and proteases.

The manuscript repeatedly claims that this is the "only reported low-cost liquid-phase assay validated specifically for comparative analysis of total protease activity in P. aeruginosa." However, liquid milk turbidity assays have been used in various bacterial systems. This statement should be softened or supported with more literature evidence.

Line 51: "Its production in P. aeruginosa is strongly regulated by quorum sensing (QS), which links population-density cues to the expression of extracellular "public goods" (15)" this sentence need to be revised for clarity.

Lines 102-103:" These endpoint OD600 values were used to compare growth across strains under assay-matched conditions" the sentence need to be revised, perhaps referring to the protease assay "under conditions matched to those used in the protease assay"

Line 205: "This pattern is biologically consistent with the established role of QS". It's ambiguous what are the authors referring to "This patter", please clarify

Gene names need to be italicized

For supernatant testing, did the authors consider adding 0.22 micron filtration step to the centrifugation

The authors claim the assay is compatible with microplate readers, yet all testing was done using cuvettes, please clarify.

Figure 4: Regression residuals should be shown

Table 4: Skim-milk agar clearing is referenced as "this study" but it was never listed and reported among the results. Please revise and/or clarify.

The manuscript by Enríquez-Ríos et al “Liquid-phase milk turbidimetry enables rapid phenotyping of secreted protease activity in Pseudomonas aeruginosa at less than one cent per sample” introduces a cost-effective, liquid-phase skim milk turbidimetry assay designed to quantify total secreted protease activity in *Pseudomonas aeruginosa*. The manuscript offers a practical low coast tool using straightforward, accessible, and reproducible protocol. The text is well-structured, clearly articulated, and fills a notable methodological gap. However, to maximize the manuscript's clarity and impact, several key areas require attention

**Major comments:**

The authors state “A600 differences were interpreted without additional OD normalization, given that endpoint OD600 was statistically indistinguishable across strains.” OD600 ≈ 3.4–4.0 represents nearly 20% variation in biomass and such small difference in culture density can influence secreted enzymes and supernatant activity. A more rigorous approach should be included (at least a sensitivity analysis showing that normalization does not change strain ranking).

Can the authors clarify why low-activity strains show higher variability.

The author’s claim of the broad applicability of the assay, this is not fully supported with only five strains are tested. While the panel includes reference strains and one QS mutant, the manuscript claims broad applicability (“diverse strain backgrounds”). Additional isolates, such as clinical isolates known with loss-of-function mutations in QS and/or QS mutants beyond *lasR/rhlR*, or hyperproteolytic variants would strengthen the generalizability of the assay.

The assay uses a commercial milk powder with collagen additives; the authors need to justify the choice of this specific milk product and include a cautionary note in the method section. Other milk brands and lots should be tested to address the milk composition and how it varies across brands and lots.

Have the authors taken into consideration the collagen additives and the possibility of these peptides interfering with turbidity or protease susceptibility?

What is the rationale of autoclaving milk? This process can denature proteins in inconsistent ways.

The manuscript does not validate which proteases contribute most to the signal. For example, LasB, AprA, LasA, and PIV have different substrate preferences, it is difficult to interpret the biological meaning of the A600 readout. Did the authors consider using specific inhibitor studies and/or knockout panels. The manuscript would benefit from at least one validation experiment.

The manuscript reports means and SDs from n = 3 biological replicates but no technical replicates are mentioned.
Given the small sample size, the authors should justify the statistical approach or provide more sample size

The authors are emphasizing the intralaboratory reproducibility, yet all data are reported from a single laboratory. For a method paper, cross-lab validation is highly desirable. Even a small two-lab comparison would significantly increase confidence in the assay's robustness.

In the discussion section, the comparison to elastin–Congo red and azocasein assays is strong, but unless sufficient evodence are provided, the authors should avoid implying that milk turbidity is inherently more "biologically relevant".

Minor comments:

Line 11-13: This sentence is correct but overly long. Consider splitting for clarity: "Secreted proteases are major virulence determinants in *Pseudomonas aeruginosa* and vary widely across strains and quorum-sensing (QS) backgrounds, thereby motivating the development of simple, standardized phenotyping tools."

The Introduction is overly long and repeat background information on QS and proteasas.

The manuscript repeatedly claims that this is the "only reported low-cost liquid-phase assay validated specifically for comparative analysis of total protease activity in *P. aeruginosa*." However, liquid milk turbidity assays have been used in various bacterial systems. This statement should be softened or supported with more literatura evidence

Line 51: "Its production in *P. aeruginosa* is strongly regulated by quorum sensing (QS), which links population-density cues to the expression of extracellular "public goods" (15)" this sentence need to be revised for clarity.

Lines 102-103:" These endpoint OD600 values were used to compare growth across strains under assay-matched conditions" the sentence need to be revised, perhaps referring to the prease assay "under conditions matched to those used in the protease assay"

Line 205: "This pattern is biologically consistent with the established role of QS". It's ambigious what are the authors referring to "This patter", please calrify

Gene names need to be italicized

For supertant testing, did the authors consider adding 0.22 micron filteration step to the centrifigation

The autors claim the assay is compatible with microplate readers, yeta ll testing was done using cuvettes, please calrify.

Figure 4: Regression residuals should be shown

Table 4: Skim-milk agar clearing is referenced as “this study” but it was never listed and reposrted among the results. Please revise and/or clarify.

We thank the Editor and the Reviewers for their careful evaluation of our manuscript and for their constructive comments and suggestions. We have carefully considered all observations and have revised the manuscript accordingly. Below, we provide a detailed, point-by-point response to each comment.

| **Reviewer #1** | **Answer:** | **Lines** |
|---|---|---|
| Line 43: Please remove the period between "biology" and reference (9). | The period has been removed. | Done |
| Line 48: The sentence ending with "inference" appears to lack an appropriate supporting reference. Please add the relevant citation. | We have added supporting references:<br><br>Galdino ACM, Branquinha MH, Santos ALS, Viganor L. 2017. *Pseudomonas aeruginosa* and its arsenal of proteases: Weapons to battle the host, p. 381–197. *In* Chakraborti S., DN (ed.), Pathophysiological Aspects of Proteases. Springer, Singapore.<br><br>Kessler E, Safrin M. 2014. Elastinolytic and proteolytic enzymes. Methods in Molecular Biology 1149:135–169.<br><br>to substantiate the statement that total secreted protease activity is commonly used as an integrative functional phenotype for comparing strains and evaluating regulatory mechanisms in *P. aeruginosa*. | Lines: 50, 52, 58<br>Lines:58, 105,110 |
| Line 51: The first part of the sentence be rephrased to: The production of these proteases in...". | We have revised the sentence as recommended to improve clarity and avoid ambiguity regarding the antecedent of "its production." The sentence now reads: "The production of these proteases in *P. aeruginosa* | Lines 60-61 |

| | | |
|---|---|---|
| | is strongly regulated by quorum sensing (QS), which links population-density cues to the expression of extracellular 'public goods' (15)." | |
| Line 93: Please add "See Table 1" following "ATCC 9027". | We have added a reference to Table 1 following the mention of ATCC 9027 to facilitate identification of the strains included in the study. | Line: 146 |
| Line 113: The skim milk solution and assay buffer were autoclaved at 121 °C for 15 min. Have the authors considered whether this treatment could alter the milk proteins and affect assay performance? Please also discuss whether heat-induced changes in skim milk components, such as casein-related structure or other protein denaturation, could influence reproducibility or comparability of the assay. | We agree that autoclaving can induce structural changes in milk proteins, particularly denaturation of whey proteins and alterations in casein micelle organization. However, sterilized skim milk is routinely used as a substrate in milk agar, where the milk-containing medium is typically autoclaved prior to use. Because all substrate preparations in our study were subjected to the same sterilization procedure, any heat-induced modifications were constant across experiments and therefore unlikely to affect relative comparisons among strains. To address this point, we have added a statement in the Methods/Discussion acknowledging that autoclaving may alter milk protein structure and that substrate preparation should be standardized to ensure reproducibility and comparability across laboratories. | Lines 178-184<br>Lines 749-762 |
| Lines 124-127: In Conditions A and B, different volumes of | The reviewer is correct that the skim milk substrate was prepared in water | Line: 202, 205<br>Line 736-742 |

| | | |
|---|---|---|
| milk substrate and assay buffer are used. As a result, the final concentrations of assay components, including Tris and $CaCl_2$, may differ between the two conditions. Please clarify whether this could introduce bias into the comparison, particularly if the higher $CaCl_2$ concentration in Condition B may influence protease activity or assay readout independently of the milk load | rather than in assay buffer. Consequently, the final concentrations of Tris-HCl and $CaCl_2$ differed slightly between the two substrate-load conditions. Specifically, Condition A (100 µL milk substrate + 800 µL assay buffer) contained **80 mM Tris-HCl and 0.8 mM $CaCl_2$**, whereas Condition B (250 µL milk substrate + 650 µL assay buffer) contained **65 mM Tris-HCl and 0.65 mM $CaCl_2$.** We acknowledge that this represents a minor difference in reaction composition. However, **both conditions remain within ranges commonly employed for extracellular protease assays**. For example, Kessler and Safrin (2014) describe azocasein and elastin–Congo red assays for *P. aeruginosa* using reaction buffers containing 50 mM Tris-HCl and 0.5 mM $CaCl_2$. Therefore, the Tris-HCl and $CaCl_2$ concentrations used in both of our assay conditions are comparable to those reported for established extracellular protease assays. Importantly, despite these modest differences in buffer composition, both substrate-load conditions produced highly consistent results, preserving strain ranking and showing a strong correlation between mean activity values. | |

| | | |
|---|---|---|
| | This indicates that the observed differences between Conditions A and B were driven by substrate load rather than by the small differences in Tris-HCl or $CaCl_2$ concentrations.<br>To address this point, we have added a clarification in the methods and discussion sections. | |
| Table 2: The turbidity values (A600) for both the 2 mg and 5 mg conditions are already presented in Figures 2 and 3. As a result, Table 2 partly repeats information that is already shown elsewhere, with CV% being the main additional value. The authors may restructure the presentation to reduce redundancy, for example by simplifying the table, integrating only the variability data, or moving part of the material to the Supplementary Information. | We agree that Figures 2 and 3 illustrate the strain-dependent trends in residual turbidity. However, we believe that Table 2 provides complementary information by presenting the exact quantitative values (mean ± SD) together with the corresponding coefficients of variation, allowing readers to directly evaluate both assay performance and reproducibility. The figures facilitate visual comparison and statistical interpretation, whereas the table serves as a quantitative reference. To better emphasize this distinction, we have modified the title of Table 2 to explicitly indicate that it **summarizes both the quantitative A600 measurements and the intralaboratory reproducibility of the assay** “Table 2. Quantitative residual turbidity (Mean A600 ± SD) and intralaboratory reproducibility (CV%) of the liquid-phase milk turbidimetry assay under | Line 406 |

| | | |
|---|---|---|
| | the 2 mg and 5 mg substrate-load conditions."<br><br>In addition, we have modified the column headings to specify Mean A600 ± SD instead of simply Turbidity A600, making the purpose of the table clearer. | |
| Figures 2 and 3: The authors should explain more clearly the behavior of strain DVL1758 across the two milk substrate loads. In the 2 mg condition (Figure 2), DVL1758 appears not to differ clearly from the LB control, the mutant, and ATCC 9027, whereas in the 5 mg condition (Figure 3) it is grouped together with PAO1 and PA14 and separated from the low-activity group. This shift in statistical grouping is important for interpretation and deserves clearer explanation in the Results or Discussion. | We agree that the behavior of DVL1758 deserves further clarification. Although DVL1758 exhibited an intermediate proteolytic phenotype under both substrate-load conditions, its statistical grouping differed because the higher milk load improved separation among strains. At 2 mg, DVL1758 showed lower residual turbidity than the low-protease group but remained intermediate between the highly proteolytic strains (PAO1 and PA14) and the low-activity strains, resulting in less distinct statistical separation. At 5 mg, the increased substrate load enhanced signal stability and allowed DVL1758 to cluster with PAO1 and PA14 while remaining clearly separated from the low-activity strains (this data is consistent with other reports (García-Reyes et al. 2025 **doi: 10.1093/femsml/uqaf031**). Importantly, the overall strain ranking was preserved across both conditions, indicating that the observed change reflects improved statistical | Lines 295-298<br>Lines: 350-358 |

| | | |
|---|---|---|
| | resolution rather than a shift in biological phenotype. We have added text in the Results section to clarify this point. | |
| Lines 196 and 226: Please double-check the table citations in the Results section. It is not clear whether the manuscript should refer to Table 1 (strain information) in these two lines. | We reviewed the table citations in the Results section and confirmed that the reference to Table 1 in these instances was incorrect. Table 1 contains strain information and is not directly related to the assay performance metrics discussed in these paragraphs. The table citations have been corrected; it must be Table 2. | Line: 397 |
| Figure 4: In the figure legend, the authors state that the association becomes stronger when the LB control is excluded. However, the figure itself appears to show only the analysis including the LB control. It would be more reasonable to present both versions, with and without the LB control. | Figure 4 presents the complete dataset, including the LB negative control, which is explicitly identified as the red data point. The statement regarding exclusion of the LB control refers to the complementary statistical analysis performed on the same dataset after removing this single point, rather than to a separate experimental dataset. Because excluding the LB control simply recalculates the correlation using the same data, we believe that adding a second panel would be redundant and would not provide additional biological information. To improve clarity, we have revised the figure legend to explicitly indicate that the red point corresponds to the LB negative control and that the statistics obtained after excluding this point are reported as | Line: 491, 493-496 |

| | | |
|---|---|---|
| | complementary information. | |
| Line 310: The manuscript states that CV < 20% indicates acceptable precision. Please clarify the basis for this criterion and provide an appropriate reference to support it. | The statement was based on commonly accepted bioanalytical method validation criteria, where intra-assay precision is generally considered acceptable when the coefficient of variation does not exceed 15–20%, depending on the assay context and concentration range.<br>We agree that this criterion should be explicitly supported. Accordingly, we have revised the text and added references to the FDA Bioanalytical Method Validation Guidance (2018) and the EMA Guideline on Bioanalytical Method Validation (2022), which use similar precision benchmarks for assay validation. | Line: 505 |
| Table 4, Readout column: Please explain the meaning of the symbols used in the Readout column, such as the up arrow, down arrow, and equals sign. Their interpretation is not currently defined, which may make the table unclear to readers. | We agree that the symbols used in the Readout column should be explicitly defined. We have therefore added a footnote to Table 4 clarifying that, in the liquid-phase milk turbidimetry assay, a decrease in A600 (↓) reflects increased proteolytic activity due to substrate degradation and loss of turbidity. In contrast, for azocasein, elastin–Congo red, and fluorogenic substrate assays, an increase in absorbance or fluorescence (↑) reflects increased proteolytic activity. This clarification has been added to improve table readability. | Line: 579-581 |

| | | |
|---|---|---|
| Table 4, References column: For the skim-milk agar clearing method, the reference is given as "this study." If this technique is already established and only discussed or used here for comparison, the authors should cite the original article(s) describing or applying this method. | We agree that the skim-milk agar clearing assay is a well-established method and should not be cited as "This study." We have revised Table 4 to replace "This study" with the reference: **Pseudomonas methods and protocols** Cadoret F, Soscia C, Voulhoux R*New York, Humana Press, New York, NY, (2014), 1, 271-279*<br><br>This describing the use of skim-milk agar for the detection of extracellular protease activity in P. aeruginosa and related bacteria. | Line 578, Table 4 |
| Lines 365 and 401: Please check the consistency of table numbering throughout the manuscript. Table 6 appears to be mentioned earlier in the text, whereas the first mention of Table 5 occurs later. The order of table citations should be made fully consistent. | Thank you for identifying this issue. We reviewed all table citations throughout the manuscript and confirmed that Table 5 was not explicitly introduced before subsequent table references. We have revised the text to cite Table 5 in the appropriate section immediately before its presentation, ensuring that all tables are now cited sequentially according to their order of appearance in the manuscript. | Lines:540, 572 |

| **Reviewer #2** | **Answer:** | **Lines** |
|---|---|---|
| The authors state "A600 differences were interpreted without additional OD normalization, given that endpoint OD600 was statistically indistinguishable across | We agree that differences in culture density can potentially influence the amount of secreted enzymes recovered from culture supernatants. In the original manuscript, | Line 449 |

| | | |
|---|---|---|
| strains." OD600 ≈ 3.4-4.0 represents nearly 20% variation in biomass and such small difference in culture density can influence secreted enzymes and supernatant activity. A more rigorous approach should be included (at least a sensitivity analysis showing that normalization does not change strain ranking) | additional normalization was not performed because endpoint OD600 values did not differ significantly among strains (Figure 1), indicating that all cultures reached comparable cell densities under identical growth conditions.<br><br>Nevertheless, we appreciate the suggestion and performed an additional **sensitivity analysis** by expressing the residual turbidity values relative to the corresponding endpoint culture OD600 for each strain.<br><br>**This sensitivity analysis demonstrated that normalization did not alter the overall interpretation of the results.** Under both the 2 mg and 5 mg substrate-load conditions, the assay continued to discriminate between high and low-protease phenotypes. Therefore, the modest differences in endpoint culture density did not affect the biological conclusions of the study.<br><br>To address the reviewer's concern, we have included this sensitivity analysis a Table 3. These additional data further support the robustness of the assay and | |

| | | |
|---|---|---|
| | indicate that the observed differences in protease activity are not driven by small variations in culture biomass. | |
| -Can the authors clarify why low-activity strains show higher variability. | Higher variability in the low-activity strains is primarily explained by the fact that these samples produced only minimal changes in milk turbidity. Under these conditions, small experimental fluctuations (for example: pipetting variability, minor differences in substrate dispersion, or instrumental noise) represent a larger proportion of the measured signal, resulting in higher coefficients of variation (CV). This behavior is expected for turbidity-based assays operating near the lower limit of the analytical response and does not indicate poor assay performance. Importantly, despite the slightly higher CV values observed for low-protease strains, all coefficients of variation remained below 20%, and these strains were consistently distinguished from the high-protease phenotypes across independent experiments. We have clarified this point in the Discussion part. | Line 666-680 |

| | | |
|---|---|---|
| -The author's claim of the broad applicability of the assay, this is not fully supported with only five strains are tested. While the panel includes reference strains and one QS mutant, the manuscript claims broad applicability ("diverse strain backgrounds"). Additional isolates, such as clinical isolates known with loss-of-function mutations in QS and/or QS mutants beyond lasR/rhlR, or hyperproteolytic variants would strengthen the generalizability of the assay. | We agree that the strain panel evaluated in this study does not encompass the full genetic and phenotypic diversity of *P. aeruginosa*. Our objective was to validate the assay using a representative set of strains with well characterized and contrasting proteolytic phenotypes, including the laboratory reference strains PAO1 and PA14, a QS-deficient mutant (PAO1Δ*lasR*Δ*rhlR*), an environmental isolate (DVL1758), and a clinical isolate with reduced total protease production (ATCC 9027). This panel allowed us to evaluate the assay across a range of extracellular protease activities and regulatory backgrounds.<br><br>We agree, however, that broader validation using larger collections of clinical and environmental isolates, including naturally occurring QS-deficient strains, additional regulatory mutants, and hyperproteolytic variants, would further strengthen the generalizability of the assay. Accordingly, we have moderated the wording throughout the manuscript and now identify broader validation across a more diverse collection of | Lines: 126-127<br>Lines:781-785 |

| | | |
|---|---|---|
| | isolates as an important direction for future work.<br><br>We change: “across diverse strain backgrounds” by: “applicable to strains with distinct proteolytic phenotypes” | |
| -The assay uses a commercial milk powder with collagen additives; the authors need to justify the choice of this specific milk product and include a cautionary note in the method section. Other milk brands and lots should be tested to address the milk composition and how it varies across brands and lots. | We agree that the composition of commercial skim milk powders may vary among manufacturers and even between production lots, potentially affecting assay performance. The commercial skim milk powder used in this study was selected because it was readily available, inexpensive, and produced reproducible results throughout the validation experiments. Although this product contains added collagen, the assay is intended to measure the overall degradation of a complex protein substrate rather than the activity of a specific protease, and the same substrate preparation was used consistently for all experiments.<br>We acknowledge, however, that differences in protein composition among commercial milk products could influence absolute turbidity values. **Therefore, we have added a cautionary statement in the Materials and** | Lines: 169-176<br>Lines 773-778 |

| | | |
|---|---|---|
| | **Methods and Discussion** indicating that substrate source and preparation should be standardized within a study, and that validation using additional milk brands would be an important direction for future work. | |
| -Have the authors taken into consideration the collagen additives and the possibility of these peptides interfering with turbidity or protease susceptibility? | We acknowledge that the commercial skim milk powder used in this study contains added collagen and that additional protein components could, in principle, influence substrate turbidity or protease susceptibility.<br><br>However, our results do not suggest that the presence of collagen introduced a detectable bias in assay performance. Specifically, strains with well-established proteolytic phenotypes behaved as expected: the QS-deficient mutant (PAO1Δ*lasR*Δ*rhlR*) displayed minimal proteolytic activity, the clinical isolate ATCC 9027 exhibited reduced activity consistent with previous reports, and the reference strains PAO1 and PA14 showed the highest levels of substrate degradation. Thus, the assay correctly reproduced the expected biological ranking despite the presence of collagen in | Lines: 169-174<br>Lines 715-716 |

| | | |
|---|---|---|
| | the commercial milk preparation.<br><br>Nevertheless, because we evaluated only a single commercial skim milk product, we cannot exclude the possibility that different formulations or additives could influence absolute assay signals. We have therefore added a cautionary statement in the Methods and Discussion indicating that the milk source should be standardized within a study and that future work should evaluate assay performance using additional commercial milk formulations. | |
| -What is the rationale of autoclaving milk? This process can denature proteins in inconsistent ways. | We agree that autoclaving can induce structural changes in milk proteins, particularly denaturation of whey proteins and alterations in casein micelle organization. However, **sterilized skim milk is routinely used as a substrate in milk agar protocol**, where the milk-containing medium is typically autoclaved before use. Because all substrate preparations in our study were subjected to the same sterilization procedure, any heat-induced modifications were constant across experiments and therefore unlikely to | Lines: 180-186<br>Lines: 759-773 |

| | | |
|---|---|---|
| | affect relative comparisons among strains. To address this point, we have added a statement in the Methods/Discussion acknowledging that autoclaving may alter milk protein structure and that substrate preparation should be standardized to ensure reproducibility and comparability across laboratories. | |
| -The manuscript does not validate which proteases contribute most to the signal.<br>For example, LasB, AprA, LasA, and PIV have different substrate preferences, it is difficult to interpret the biological meaning of the A600 readout. Did the authors consider using specific inhibitor studies and/or knockout panels. The manuscript would benefit from at least one validation experiment. | Our intention was not to develop an assay specific for any individual extracellular protease, but rather to provide a simple phenotypic assay that measures the cumulative total extracellular proteolytic activity present in liquid culture supernatants. Because skim milk contains protein substrate, the measured turbidity loss is expected to reflect the combined activity of several secreted proteases, including LasB, AprA, LasA, PIV, and potentially additional extracellular enzymes.<br><br>Defining the relative contribution of individual proteases using knockout strains or selective inhibitors would provide valuable mechanistic information. However, such experiments fall outside the scope of the present methodological study, | Lines: 52-56<br>Lines: 654-655<br>Lines: 709-710<br>Lines:736-741 |

| | | |
|---|---|---|
| | whose objective was to validate a simple comparative assay for total secreted protease activity rather than to dissect the contribution of individual proteases.<br><br>To clarify this point, we have revised the Introduction and Discussion to explicitly state that the assay provides an integrated phenotypic readout of extracellular proteolysis and should be considered complementary to targeted biochemical or genetic approaches when mechanistic resolution is required. | |
| -The manuscript reports means and SDs from n = 3 biological replicates but no technical replicates are mentioned. Given the small sample size, the authors should justify the statistical approach or provide more sample size | We apologize for the lack of clarity in the original manuscript. The study was performed using three independent biological replicates, and each biological replicate **was analyzed in triplicate technical replicates** under each assay condition. Statistical analyses were conducted using the mean value of the technical replicates for each independent biological replicate (n = 3 biological replicates). We have revised the Materials and Methods to describe the experimental design more explicitly and have also clarified this information in the corresponding figure | We have added the data in all figure descriptions when was required. |

| | | |
|---|---|---|
| | legends to avoid ambiguity. | |
| -The authors are emphasizing the intralaboratory reproducibility, yet all data are reported from a single laboratory. For a method paper, cross-lab validation is highly desirable. Even a small two-lab comparison would significantly increase confidence in the assay's robustness. | We agree that interlaboratory validation would further strengthen confidence in the robustness and transferability of the proposed assay, particularly for a methodological study.<br><br>In the present work, our validation focused on demonstrating the analytical and biological performance of the assay within a single laboratory. Specifically, the method was evaluated using strains representing well-established differences in total secreted protease activity, including reference strains, a QS double mutant, and additional environmental and clinical isolates. Furthermore, assay robustness was assessed under two independent substrate-load conditions (2 mg and 5 mg milk per reaction), both of which yielded consistent strain ranking and acceptable intralaboratory reproducibility.<br><br>We nevertheless agree that an independent interlaboratory comparison would provide an additional level of validation. We therefore intend to perform a two-laboratory | pending |

| | | |
|---|---|---|
| | evaluation. However, because the reviewers' comments were received immediately before the institutional holiday period, it was not possible to complete this additional work within the current revision deadline. We will therefore request additional time from the Editor to carry out this experiment and incorporate the results into the revised manuscript. | |
| In the discussion section, the comparison to elastin-Congo red and azocasein assays is strong, but unless sufficient evidence are provided, the authors should avoid implying that milk turbidity is inherently more "biologically relevant". | We agree that our data do not demonstrate that liquid-phase milk turbidimetry is inherently more biologically relevant than established assays such as azocasein or elastin–Congo red. Our assay provides an integrated measure of extracellular proteolytic activity using a complex natural substrate, rather than the activity of a single protease or substrate-specific reaction. **To avoid overinterpretation**, we have revised the Discussion part to remove statements implying superior biological relevance and instead describe the assay as a complementary approach with distinct practical advantages and limitations relative to existing methods. | Lines: 703-705 |
| Line 11-13: This sentence is correct but | We agree that the original sentence was | Lines: 11-14 |

| | | |
|---|---|---|
| overly long. Consider splitting for clarity: "Secreted proteases are major virulence determinants in Pseudomonas aeruginosa and vary widely across strains and quorum-sensing (QS) backgrounds, thereby motivating the development of simple, standardized phenotyping tools." | unnecessarily long and could be improved for readability. We have revised the text by splitting it into two shorter sentences while preserving its original meaning. | |
| The Introduction is overly long and repeat background information on QS and proteases. | In the revised manuscript, we have streamlined this section by removing redundant background information, reducing overlap between paragraphs, and focusing more directly on the rationale for developing a simple, quantitative assay for comparative analysis of total secreted protease activity. These revisions improve the overall flow and readability of the Introduction while preserving the essential biological context. | done |
| The manuscript repeatedly claims that this is the "only reported low-cost liquid-phase assay validated specifically for comparative analysis of total protease activity in P. aeruginosa." However, liquid milk turbidity assays have been used in various bacterial systems. This statement should be softened or supported with more literature evidence. | We emphasize that we are not aware of a standardized liquid-phase milk turbidimetry assay that has been specifically developed and validated for comparative analysis of total secreted protease activity in *P. aeruginosa*. To avoid overstating the novelty of the method, we have revised the manuscript throughout by softening this claim and clarifying that the novelty lies in the | Lines: 645-648 |

| | | |
|---|---|---|
| | adaptation, validation, and benchmarking of the assay for comparative protease phenotyping in *P. aeruginosa*. | |
| Line 51: "Its production in P. aeruginosa is strongly regulated by quorum sensing (QS), which links population-density cues to the expression of extracellular "public goods" (15)" this sentence need to be revised for clarity | We agree that the original sentence could be expressed more clearly. We have revised the text to explicitly refer to extracellular protease production and to improve the overall readability of the sentence while preserving its original meaning. | Lines 60-62 |
| Lines 102-103:" These endpoint OD600 values were used to compare growth across strains under assay-matched conditions" the sentence need to be revised, perhaps referring to the protease assay "under conditions matched to those used in the protease assay" | The sentence has been revised to explicitly indicate that endpoint growth measurements were obtained under the same experimental conditions used for the protease assay. This clarification has been incorporated in the **Materials and Methods (Bacterial growth assay)** section and is further reinforced in the **Results** section describing the growth comparison among strains. | Lines: 157-159<br>Lines: 255-256 |
| Line 205: "This pattern is biologically consistent with the established role of QS". It's ambiguous what are the authors referring to "This patter", please clarify | We have revised the sentence to explicitly refer to the observed strain-dependent differences in milk clearance and the reduced proteolytic phenotype of the QS mutant, thereby improving clarity. | Lines: 306-308 |
| Gene names need to be italicized | We have carefully revised the manuscript and italicized all bacterial gene names | Done |

| | | |
|---|---|---|
| | throughout the text, tables, and figure legends in accordance with standard genetic nomenclature, while retaining protein names and strain designations in roman type. | |
| For supernatant testing, did the authors consider adding 0.22 micron filtration step to the centrifugation | We agree that 0.22 µm filtration is an effective approach to obtain cell-free supernatants. In this study, however, culture supernatants were prepared by centrifugation only, as our aim was to establish a simple and low-cost workflow using a procedure commonly employed for extracellular enzyme assays.<br>Although we did not directly evaluate residual bacterial carryover after centrifugation, our results suggest that any carryover was unlikely to have substantially influenced the assay outcome. Notably, the QS-deficient strain, which was processed identically to all other strains, consistently exhibited minimal milk degradation under both substrate-load conditions. If residual bacteria had made a major contribution to the observed proteolytic activity during the assay, a greater degree of milk degradation would also be expected in this mutant. | Lines 745-751 |

| | | |
|---|---|---|
| | To clarify this limitation, we have added a statement in the Discussion indicating that future studies may incorporate a 0.22 μm filtration step when completely cell-free supernatants are required, although the comparative results obtained here were internally consistent using centrifugation alone. | |
| The authors claim the assay is compatible with microplate readers, yet all testing was done using cuvettes, please clarify. | We agree that the assay was experimentally validated using cuvette-based spectrophotometric measurements and not in a microplate format. Our intention was to indicate that the assay principle is readily adaptable to standard microplate readers because it relies on a simple absorbance measurement at 600 nm. To avoid overstating our findings, we have revised all manuscript throughout by replacing statements referring to microplate compatibility with wording indicating that the assay is amenable to or readily adaptable *to* microplate-based implementation. We also clarify that formal validation in a microplate format remains an important objective for future work. | Lines: 16-17<br>Lines: 119-121<br>Lines: 207-210<br>Lines: 783-786 |

| | | |
|---|---|---|
| Figure 4: Regression residuals should be shown | We agree that examination of regression residuals is generally useful when evaluating linear regression models. However, the analysis presented in Figure 4 was intended as a complementary assessment of the consistency between the two substrate-load conditions rather than as a predictive regression model. Because the analysis is based on a small number of observations (six strains, or five when the LB control is excluded), formal residual diagnostics provide limited additional information and are difficult to interpret robustly. We have therefore retained the original figure but clarified in the manuscript that the regression is presented solely to illustrate the strong concordance between the two assay conditions rather than to establish a predictive relationship. | Lines: 464-466 |
| Table 4: Skim-milk agar clearing is referenced as "this study" but it was never listed and reported among the results. Please revise and/or clarify. | We agree that the skim-milk agar clearing assay is a well-established method and should not be cited as "This study." We have revised Table 4 to replace "This study" with the reference: Pseudomonas methods and protocols Cadoret F, Soscia C, Voulhoux R*New York, Humana* | Now it is named as Table 5. Reference 30. |

| | | |
|---|---|---|
| | *Press, New York, NY, (2014), 1, 271-279* | |

Re: Spectrum01271-26R1 (**Liquid-phase milk turbidimetry enables rapid phenotyping of secreted protease activity in *Pseudomonas aeruginosa* at less than one cent per sample**)

Dear Dr. Selene García-Reyes:

Your manuscript has been accepted, and I am forwarding it to the ASM production staff for publication. Your paper will first be checked to make sure all elements meet the technical requirements. ASM staff will contact you if anything needs to be revised before copyediting and production can begin. Otherwise, you will be notified when your proofs are ready to be viewed.

**Data Availability:** ASM policy requires that data be available to the public upon online posting of the article, so please verify all links to sequence records, if present, and make sure that each number retrieves the full record of the data. If a new accession number is not linked or a link is broken, provide production staff with the correct URL for the record. If the accession numbers for new data are not publicly accessible before the expected online posting of the article, publication may be delayed; please contact ASM production staff immediately with the expected release date.

**Publication Fees:** For information on publication fees and which article types have charges, please visit our website. We have partnered with Copyright Clearance Center (CCC) to collect author charges. If fees apply to your paper, you will receive a message from no-reply@copyright.com with further instructions. For questions related to paying charges through RightsLink, please contact CCC at ASM_Support@copyright.com or toll free at +1-877-622-5543. CCC makes every attempt to respond to all emails within 24 hours.

**ASM Membership:** Corresponding authors may join or renew ASM membership to obtain discounts on publication fees. Need to upgrade your membership level? Please contact Customer Service at Service@asmusa.org.

**Embargo Policy:** A press release may be issued as soon as the manuscript is posted on the Spectrum Latest Articles webpage. The corresponding author will receive an email with the subject line "ASM Journals Author Services Notification" when the article is available online.

**Cover Image Submissions:** If you would like to submit a potential Cover Image, please email a file and a short legend to spectrum@asmusa.org. Please note that we can only consider images that (i) the authors created or own and (ii) have not been previously published. By submitting, you agree that the image can be used under the same terms as the published article. Image File requirements: TIF/EPS, 7.5 inches wide by 8.25 inches tall (at least 2,250 pixels wide by 2,475 pixels tall), minimum 300 dpi resolution (600 dpi preferred), RGB, and no figure elements, e.g., arrows or panel labels. The legend should be a short description of the image, 1-2 sentences recommended. Please use the ASM Journals Cover Image Sizing Tool (https://journals.asm.org/pb-assets/html/asm-cover-sizing-tool.html) to ensure that your proposed cover image meets our size requirements.

The ASM Journals program strives for constant improvement in our submission and publication process. Please tell us how we can improve your experience by taking this quick Author Survey.

Thank you for submitting your paper to Spectrum.

Sincerely,
Sadjia Bekal
Editor
Microbiology Spectrum

Reviewer #1 (Comments for the Author):

Thank you for the revised manuscript and for careful attention to my comments. I appreciate the revisions made, which have addressed the points I raised satisfactorily.I have no further comments.

Reviewer #2 (Comments for the Author):

Comments have been addressed
